# Inhibition of FKBP5 alleviates obstetric antiphospholipid syndrome by regulating macrophage polarization

**DOI:** 10.1038/s41598-025-25472-9

**Published:** 2025-11-21

**Authors:** Mingjie Song, Juan Wang, Wenli Mu, Yanwei Zheng, Rongzhen Jiang, Yanjun Cai, Yincheng Teng, Yu Xia

**Affiliations:** 1 Department of Obstetrics and Gynecology, Shanghai Sixth People’s Hospital Affiliated to Shanghai Jiao Tong University School of Medicine, 600 Yishan Road, Shanghai, 200233 China; 2 Central Laboratory, Shandong Provincial Hospital Affiliated to Shandong First Medical University, 324 Jingwu Road, Jinan, 250021 Shandong China; 3 Department of Obstetrics, Shandong Provincial Hospital Affiliated to Shandong First Medical University, 324 Jingwu Road, Jinan, 250021 Shandong China

**Keywords:** FKBP5, Obstetric antiphospholipid syndrome, Macrophage polarization, JAK/STAT, Trophoblast, RNA, Risk factors

## Abstract

Immune homeostasis disturbance within the placental microenvironment plays a key role in the pathogenesis of obstetric antiphospholipid syndrome (OAPS), although the underlying mechanisms remain poorly understood. This study aimed to investigate the immune regulatory role of FK506-binding protein 5 (FKBP5) in OAPS-associated pathological pregnancies. RNA sequencing and immunocytotyping were performed on decidual tissues from OAPS patients and healthy controls. Bone marrow-derived macrophages (BMDMs) polarization assays and macrophage‒trophoblast coculture models were employed to explore the effects and mechanisms of FKBP5 on macrophage polarization at the maternal–fetal interface. We applied an animal model of OAPS and comprehensively evaluated the therapeutic effects of the FKBP5 inhibitor SAFit2 on OAPS. The results revealed significantly greater expression of FKBP5 and inflammation-related factors in OAPS patients than in healthy controls. An imbalance in macrophage polarization was observed, with an increase in M1 (iNOS^+^/CD86^+^) macrophages and a decrease in M2 (Arg-1^+^/CD206^+^) macrophages in OAPS patients. In vitro studies demonstrated that FKBP5 may promote M1 polarization via the JAK1/STAT1 pathway and may inhibit M2 polarization through the PPARγ/STAT6 pathway. FKBP5-induced aberrant macrophage polarization impaired trophoblast migration, invasion, and proliferation. In vivo, FKBP5 knockdown in mice alleviated aPLs-induced placental injury, facilitated epithelial‒mesenchymal transition (EMT), and restored the M1/M2 macrophage balance. Furthermore, SAFit2 ameliorated pregnancy complications in the mouse OAPS model. FKBP5 regulates immune dysfunction associated with OAPS by modulating macrophage polarization, and its inhibition could mitigate aPLs-induced placental injury. These findings suggest that FKBP5 may serve as a promising therapeutic target for OAPS.

**Supplementary Information:**

The online version contains supplementary material available at 10.1038/s41598-025-25472-9.

## Introduction

Obstetric antiphospholipid syndrome (OAPS), a systemic autoimmune disease driven by persistent antiphospholipid antibodies (aPLs), is pathologically linked to severe gestational complications such as recurrent pregnancy loss, preeclampsia, and fetal growth restriction^1^. Its global incidence has risen to 1–5% of the pregnant population, with an average annual growth rate of 3.8%, posing a serious threat to the health of mothers and children^2^. aPLs are a group of autoantibodies that target phospholipid antigens, mainly including anticardiolipin (ACA), lupus anticoagulant (LAC), and anti-β_2_-glycoprotein 1 (anti-β_2_GP1) antibodies. Among these, anti-β_2_GP1 antibodies are the principal antibodies^3^. The risk of recurrent miscarriage, especially early miscarriage, in patients with anti-β2GP1 antibody-positive OAPS is about 54%, and the risk of fetal growth restriction is 15–30%, and the incidence is increasing^4^.

The standard treatment for OAPS is antithrombotic therapy, which typically involves the combination of aspirin and low-molecular-weight heparin. However, this treatment remains ineffective in approximately 30% of patients^3,5^, highlighting the urgent need for alternative therapeutic options. Initially, the pathogenetic basis of OAPS was thought to be due primarily to placental thrombosis resulting from aPLs-induced coagulation abnormalities^6^. Nevertheless, clinical observations indicate that the incidence of placental thrombosis in OAPS patients is relatively low. Instead, the placentas of most affected individuals exhibit significant immune cell infiltration^7,8^, suggesting that the pathological effects of aPLs in OAPS may be driven predominantly by immune mechanisms. This finding supports the notion that antithrombotic therapy alone may not sufficiently address the poor pregnancy outcomes associated with refractory OAPS.

Macrophage phenotype and function show significant dynamic changes at different stages of pregnancy: at the preimplantation stage, macrophages show an initial polarization shifting toward an M1-like phenotype; with the initiation of the embryo implantation process, macrophages gradually transform into a mixed M1 and M2 state. When spiral artery remodeling is complete, M2-like macrophages become a major population^9^. It has been shown that the precise regulation of M1/M2-like polarization homeostasis is closely related to the process of trophoblast cell invasion and spiral artery remodeling, and is critical to ensure the success of embryo implantation^10,11^. However, aPLs can disrupt this balance through a variety of molecular mechanisms. It has been demonstrated that aPLs may promote trophoblast cells to secrete more cytokines and chemokines, such as IFN-γ and TNF-α, through the TLR4/MyD88 pathway, which induces aberrant polarization of M1-like macrophages, and mediates an imbalance of the immune homeostasis at the maternal–fetal interface^12,13^. Meanwhile, disturbed immune homeostasis of the decidual membrane and imbalanced M1/M2-like polarization phenotype in patients with OAPS may cause apoptosis and impair the invasive capacity of extravillous trophoblast cells through the ROS/NF-κB signaling pathway, leading to abnormal spiral artery remodeling^14^. The complex dialogue between functional cells at the maternal–fetal interface is still tightly regulated by many unknown signaling factors^15^, and the disruption of the maternal–fetal interface microenvironment and its intercellular crosstalk induced by the impairment of immune cell function may be an important immune basis for the pathogenesis of OAPS.

Our transcriptome analysis of patients with OAPS revealed upregulated FK506 binding protein 5 (FKBP5) expression in the decidual tissue of these patients. FKBP5 gene encodes an important immune-associated protein that contributes to a variety of physiological and pathological functions, including immunomodulation, protein folding, trafficking, and steroid hormone receptor administration^16^. In our earlier research, we showed that FKBP5 not only regulates the progression of various sex hormone-dependent tumors^17–19^ but is also closely associated with the immune regulation of the reproductive system. For example, FKBP5 increases uterine tolerance to improve the success of embryo transfer by regulating the decidualization of endometrial stromal cells^20^ and promotes osteoclast differentiation, which is implicated in hormone-dependent osteoporosis^21^. Recently the role of FKBP5 in the damage of placental origin pathological pregnancy has attracted much attention. It has been reported that FKBP5 is highly expressed in placental tissues of patients with recurrent miscarriage and is able to regulate trophoblast function through the PI3K/AKT signaling pathway^22^. These provide evidence that FKBP5 regulates pregnancy-related diseases.

Currently, hydroxychloroquine (HCQ) is being used experimentally in the clinical treatment of refractory OAPS based on its anti-inflammatory and immunomodulatory pharmacological mechanisms^23^. Additionally, various targeted inhibitors, including glucocorticoids, immunosuppressants, intravenous immunoglobulins (IVIGs), pravastatin, complement inhibitors, and antibodies, are being developed and tested^24,25^. FKBP5, a glucocorticoid receptor that can bind to immunosuppressive agents such as tacrolimus (FK506) and cyclosporin A, has emerged as a promising therapeutic target in the fields of cancer, psychiatric disorders, and metabolic diseases^26,27^. While previous studies have suggested a link between FKBP5 and immune regulation in pregnancy disorders, our study is the first to demonstrate the direct role of FKBP5 in modulating macrophage polarization at the maternal‒fetal interface in OAPS, providing mechanistic insights into its therapeutic potential.

Based on the high expression pattern of FKBP5 in OAPS and its function in immune regulation and hormone-dependent reproductive disorders. In this study, we used the regulation of macrophage polarization by FKBP5 as an entry point to investigate its role and mechanism in aPLs-induced disorders of immune homeostasis at the maternal–fetal interface, and to assess the therapeutic potential of FKBP5 in pathological pregnancies with OAPS.

## Materials and methods

### Clinical sample collection

Approval for the study was granted by the Ethics Committee of Shandong Provincial Hospital Affiliated to Shandong First Medical University (No. 2023–201), and informed consent was secured from all participants involved. All procedures were carried out in accordance with the relevant guidelines and regulations of our university ethics committee and the Declaration of Helsinki. Human decidual tissue was obtained from patients diagnosed with OAPS (n = 19) and healthy control (HC) (n = 19) whose pregnancies were terminated for nonmedical reasons at Shandong Provincial Hospital Affiliated to Shandong First Medical University. After post dilation and curettage, the decidual tissue was immediately separated using sterile instruments to avoid contamination from placental villi and blood clots. These tissues were immersed in ice-cold sterile phosphate-buffered saline (PBS), then transported to the laboratory within two hours. A portion of the fresh decidual tissues was fixed in 4% paraformaldehyde (PFA), while the remaining samples were stored in cold Roswell Park Memorial Institute (RPMI) 1640 with 10% fetal bovine serum (FBS) and 1% penicillin–streptomycin (P/S). Patients with malignancies, infections, or other inflammation-related diseases were excluded from this research. All OAPS patients met the diagnostic criteria for antiphospholipid syndrome (APS) as outlined in the 2006 Sydney update. Healthy pregnant women with a history of at least one successful pregnancy, confirmed negative aPLs tests, and no other pregnancy-related complications were selected as the control group. Groups were also matched for age and gestational weeks to ensure comparability. Antibody levels were evaluated in both OAPS patients and HCs included in this study. Anti-β_2_GP1 antibody and ACA levels were quantified via enzyme-linked immunosorbent assay (ELISA; Lübeck, Germany), whereas LAC levels were assessed via an LAC1 screening reagent and LAC2 confirmation reagent (Siemens, Germany). Further information about all participants included in the study can be found in Table 1.

**Table 1:** Clinical and laboratory characteristics of the patients included in this study.

Characteristic	APS (n = 19)	HC (n = 19)
Maternal age (y)	31 ± 2	28 ± 3
Gestational weeks (w)	11.43 ± 1	11.71 ± 1
IgG or IgM anti-β₂GPI positive	13 (68.4%)	0
IgG or IgM ACA positive	9 (47.4%)	0
LAC positive	8 (42.1%)	0
Clinical manifestations		
Spontaneous abortions	12 (63.1%)	0
Severe pre-eclampsia	4 (21.1%)	0
Unexplained fetal deaths	2 (10.5%)	0
Foetal growth restriction	3 (15.8%)	0

APS: Antiphospholipid Antibody Syndrome, HC: Healthy Control, anti-β₂GPI: Anti-β₂ glycoprotein 1 antibody, ACA: Anticardiolipin antibody, LAC: Lupus anticoagulant.

### Animal model

The Animal Management and Ethics Committee of the Provincial Hospital of Shandong Provincial Hospital Affiliated to Shandong First Medical University granted approval for the experimental protocol employed in this study (No. 2023–137). This study was conducted in accordance with the ARRIVE guidelines and the Guide for the Care and Use of Laboratory Animals. All efforts were made to minimize the number of animals used and to reduce suffering during the experiment. C57BL/6 J (Wild-type and *Fkbp5*^*-/-*^) were obtained from Sayer (Suzhou, JS, China) Biotechnology Company. Figure S1 displays the primers used to identify FKBP5 knockout mice. All the mice were kept at the Institute of Model Animals at Shandong Provincial Hospital Affiliated to Shandong First Medical University, where they lived in a pathogen-free environment with a temperature between 20‒26 °C, humidity from 30‒37%, and a 12-h light/dark cycle; the mice had constant access to food and water. The mice weighed between 18 and 22 g. Heterozygous mice were mated at a 2:1 female-to-male ratio to generate both wild-type (WT) and FKBP5 knockout offspring for subsequent experiments. All animals were randomly assigned for use at the time of the experiment.

WT or *Fkbp5*^*-/-*^ mice were mated in a 2:1 female-to-male ratio and checked for plugs early in the morning each day; the day on which vaginal plugs was recorded as day 0.5 (E0.5). The OAPS mouse model was established by intravenous administration of anti-β2GP1 antibodies via tail vein injection in pregnant C57BL/6 mice. This well-validated experimental protocol effectively targets the placental interface to induce typical obstetric complications. Validated by prior mechanistic studies^28–31^, this model serves as a standardized platform for evaluating OAPS pathogenesis and therapeutic interventions. Briefly, normal pregnancy group was injected with NaCl (20 mg/kg) in the tail vein at gestation E0.5 and E7.5; OAPS group and and OAPS + SAFit2 group were injected with anti-β2GP1 antibody (100 μg/mouse/day) in the tail vein at gestation E0.5 and E7.5; and OAPS + SAFit2 group received daily tail vein injection of SAFit2 (20 mg/kg) from E8.5 to E13.5. SAFit2 is a sulfonamide analog with FKBP5 binding properties and a potent and highly selective FKBP5 inhibitor. All mice were euthanized and autopsied at E14.5, and the average weights of the fetuses and placentas were recorded and the fetal resorption frequency (FRF) was calculated. Subsequently, the placentas were rinsed with PBS to remove residual blood and then immersed in 4% PFA or stored at − 80 °C for subsequent experiments. All experiments were repeated at least three times to ensure the reproducibility and reliability of the data.

### Differentiation of bone marrow-derived macrophages (BMDMs)

BMDMs were isolated from the bone marrow of both *Fkbp5*^*-/-*^ and WT mice. Following euthanasia, the mice were dissected to remove the skin and muscle, enabling access to the tibia and femur. The bone marrow was flushed by slowly injecting 2‒3 ml of PBS via a 23G syringe needle. The resulting mixture was filtered through 40 μm nylon mesh and then subjected to centrifugation at 200 × g for 5 min. An erythrocyte lysis mixture was then added, and the mixture was centrifuged at 200 × g for an additional 10 min. The cells were cultured in Dulbecco’s modified Eagle’s medium (DMEM, Gibco, Grand Island, USA), which was supplemented with 10% FBS, 1% P/S and M-CSF (30 ng/mL) for 7 days at 37 °C under 5% CO₂ to induce BMDM differentiation. For M1 polarization, BMDMs were stimulated with lipopolysaccharide (LPS, 100 ng/mL) and interferon-γ (IFN-γ, 20 ng/mL), while interleukin-4 (IL-4, 20 ng/mL) and IL-13 (10 ng/mL) were used to facilitate M2 polarization.

### Flow cytometry

Following collection, human decidual tissue should be rinsed thoroughly 3–5 times with physiological saline to remove residual blood and minimize contamination. The rinsed tissue must then be immediately immersed in ice-cold sterile PBS within a specimen container and maintained under continuous cold conditions (4 °C) during transport to the laboratory for processing. Upon arrival, fresh decidual tissue undergoes mechanical dissociation in ice-cold PBS. This is followed by enzymatic digestion using a solution containing 1 mg/mL collagenase IV (Gibco, Grand Island, USA) and 0.1 mg/mL DNase I (Sigma‒Aldrich, St. Louis, USA), incubated at 37 °C for 40 min. The resulting cell suspension is filtered and mononuclear cells are subsequently enriched by density gradient centrifugation over a 50%, 45%, 35%, and 30% Percoll gradient prior to antibody staining. The obtained single-cell suspension was incubated with antibodies prepared in PBS buffer containing 2% FBS at 4 °C in the dark for 30 min. Analysis employed a BD LSR Fortessa with the following gating hierarchy: (1) FSC-A/SSC-A exclusion of debris; (2) FSC-H/FSC-A singlet discrimination; (3) CD45⁺ leukocyte gate; (4) CD14⁺ macrophage subset; (5) CD86⁺ (M1) or CD206⁺ (M2) frequencies. Full gating schematics are provided in Figure S2. For M1/M2 phenotyping: (1) M1 panel: CD45 APC (17-0459-42, eBioscience, San Diego, USA) + CD14 FITC (11-0149-42, eBioscience, San Diego, USA) + CD86 PE (12-0869-42, eBioscience, San Diego, USA); (2) M2 panel: CD45 APC + CD14 FITC + CD206 APC (17-2069-42, eBioscience, San Diego, USA).

Furthermore, in the in vitro cell experiments, primary BMDMs of mice were prepared as previously described. The phenotypes of M1/M2-type macrophages induced by BMDMs were analyzed via Flow cytometry (FCM). The staining protocol and gate-setting strategy are similar to those described above. This analysis involved the use of specific antibodies, namely, anti-F4/80 BV421 (123131, BioLegend, San Diego, USA), anti-CD11b FITC (101205, BioLegend, San Diego, USA), anti-CD86 PE (12-0862-81, Invitrogen, Carlsbad, USA), and anti-CD206 APC (17-2061-80, Invitrogen, Carlsbad, USA) antibodies, to detect distinct macrophage phenotypes.

### RNA-seq

Total RNA was extracted from decidualized tissues of the HC and OAPS groups via TRIzol reagent (Invitrogen, Carlsbad, CA, USA). Subsequently, the purity of the samples was determined by NanoPhotometer ® (IMPLEN, CA, USA). The concentration and integrity of RNA samples were detected by Agilent 2100 RNA nano 6000 assay kit (Agilent Technologies, CA, USA) . Sequencing libraries were generated using VAHTS Universal V6 RNA-seq Library Prep Kit for Illumina ® (NR604-01/02) following the manufacturer’s recommendations and index codes were added to attribute sequences to each sample. The raw data obtained were filtered to produce clean data, which were subsequently aligned with the reference genome via HISAT2 v2.1.0. 0. The number of reads for each gene in each sample was quantified via HTSeq v0.6.0. Differentially expressed genes (DEGs) were analyzed with DESeq2. Genes with *P* < 0.01 and |log2(fold change)|≥ log2(1.5) were classified as differentially expressed genes (DEGs). Next, heatmaps were created via the R package pheatmap. Hypergeometric tests were employed for Gene Ontology (GO) and Kyoto Encyclopedia of Genes and Genomes (KEGG) enrichment analyses^32^. Furthermore, gene set enrichment analysis (GSEA) was performed via the R package clusterProfiler. The accession number for the RNA-seq data reported in this paper is GSE283798.

### Cell culture and coculture system

The HTR8/SVneo cell line was sourced from the Chinese Type Culture Center. In the laboratory, the cells were maintained in RPMI-1640. This growth medium was enriched with 10% FBS and 1% P/S in an incubator at 37 °C in a 5% CO_2_ environment. Conditioned media from M1- and M2-like macrophages were collected and used to establish a coculture system with HTR8/SVneo cells.

### Protein extraction and Western blotting

Samples were lysed on ice via RIPA lysis buffer (R0020, Solarbio, BJ, China), which contained protease and phosphatase inhibitors. Total proteins were extracted, and the concentrations were measured via a BCA Quantitative Protein Kit (pc0020, Solarbio, BJ, China). Equal quantities of the proteins were exposed to 100 °C for 10 min to induce denaturation, followed by separation via electrophoresis on 10% SDS‒PAGE gels. These proteins were transferred to 0.22 μm polyvinylidene fluoride (PVDF) membranes via constant current. For blocking, the membrane was treated with 5% skim milk powder at room temperature for 1 h. Subsequently, protein bands of different molecular weights were incubated with specific antibodies overnight at 4 °C. The next day, the membranes were incubated with the relevant secondary antibody at room temperature for 1 h, and then the immunoreactive bands were visualized on a chemiluminescence visualizer (Amersham imager 680, USA) using an enhanced chemiluminescence (ECL) kit (Merck Millipore, Billerica, USA). Band intensity was quantified using ImageJ 1.54 g with background subtraction and normalization to β-actin loading controls. The antibodies used in this investigation included the following: anti-FKBP5 antibody (#12210, CST, Danvers, USA), anti-iNOS antibody (#13120, CST, Danvers, USA), anti-Arg-1 antibody (#93668, CST, Danvers, USA), anti-JAK1 antibody (#3332, CST, Danvers, USA), anti-pJAK1 antibody (#74129, CST, Danvers, USA), anti-STAT1 antibody (#9172, CST, Danvers, USA), anti-pSTAT1 antibody (AP0135, ABclonal, China), anti-STAT6 antibody (#5397, CST, Danvers, USA), anti-pSTAT6 antibody (#56554, CST, Danvers, USA), anti-PPARγ antibody (#2435, CST, Danvers, USA), and goat anti-rabbit IgG H&L (HRP; ab6721; Abcam, Cambridge, USA).

### Quantitative real-time PCR

Total RNA was isolated from samples via the use of TRIzol reagent. Reverse transcription to cDNA according to the instructions of the reverse transcription kit (AG, Code No. AG11728). SYBR Green-based qPCR was conducted using the SYBR Green Premix Pro Taq HS qPCR Kit (AG, Code No. AG11701). To evaluate the expression levels of the target genes, the data were analyzed via the 2^−ΔΔCT^ method, which included normalization against β-actin levels to ensure accurate comparisons. The specific primers used in these experiments are illustrated in Figure S1.

### Immunohistochemistry and immunofluorescence

For immunohistochemistry (IHC), the obtained tissues were first preserved with 4% PFA, embedded in paraffin, and sliced into 5 μm thick sections. These sections were dewaxed and hydrated, followed by antigen retrieval. Endogenous peroxidase activity was subsequently inhibited via the addition of 3% hydrogen peroxide (H_2_O_2_). These sections were treated with primary antibodies at 4 °C overnight after sealing. The next day they were treated with secondary antibody for 1 h at room temperature. Color development was achieved via diaminobenzidine (DAB), and after hematoxylin re-staining, the film was counterblue, dehydrated, and sealed.

For immunofluorescence (IF), samples were initially fixed with 4% PFA for 30 min, after which they were permeabilized with 0.2% Triton X-100 for an additional 30 min. Following a blocking step with 1% BSA in PBS at room temperature for 1 h, the samples were incubated overnight at 4 °C with the primary antibody. The next day, the sections were incubated with fluorescent secondary antibodies along with DAPI (d9542, Sigma‒Aldrich, St. Louis, USA) for 1 h in the dark before being imaged with a Leica TCS SP8 confocal fluorescence microscope (Leica Microsystems, Wetzlar, Germany). The antibodies utilized in this experiment included anti-iNOS (#13120, CST, Danvers, USA), anti-Arg-1 (#93668, CST, Danvers, USA), anti-FKBP5 (#27140, CST, Danvers, USA), anti-pJAK1 (PJAK1-140AP, Invitrogen, Carlsbad, USA), anti-pSTAT1 (#9167, CST, Danvers, USA), anti-pSTAT6 (#56554, CST, Danvers, USA), anti-PPARγ (#2435, CST, Danvers, USA), anti-iNOS (#95423, CST, Danvers, USA), anti-E-cadherin (#3195, CST, Danvers, USA), and anti-Vimentin (#5741, CST, Danvers, USA) antibodies.

### Cell invasion assay, wound healing test and EDU assay

For the cell invasion assay, pure matrix glue (BD, Biosciences, San Jose, USA) was diluted at a 1:8 ratio. Then, 60 μl of this mixture was placed in the upper chamber of a Transwell plate and incubated for 3‒5 h to facilitate the polymerization of the Matrigel into a gel. Subsequently, 600 μl of complete medium was added to the lower chamber, and 1 × 10^4^ cells were added to the upper chamber. After a 48-h incubation, the cells in the upper chamber were carefully removed with a cotton swab, fixed in 4% PFA for 30 min, rinsed with PBS, and then stained with 0.1% crystal violet for another 30 min. Images of the stained cells were captured via a DP Manager-70 microscope (Olympus, Japan).

For the wound healing assay, once the cells reached 90% confluence in the six-well plate, they were carefully scraped using a 200 μl pipette. Following this procedure, RPMI-1640 medium was added to the plates to maintain an optimal growth environment for the cells. Images were subsequently captured at two key time points: at the initial start of the experiment (0 h) and again after a duration of 48 h. These images were obtained via an inverted microscope manufactured by Olympus, which is based in Tokyo, Japan, to facilitate detailed observation and analysis of the cellular dynamics.

For the EDU assay, the assessment of the proliferative ability of the cells was conducted with an EdU kit (Cell-Light EdU Apollo567 In Vitro Kit, RiboBio, GD, China) in accordance with the guidelines provided by the manufacturer. Observations were captured via an inverted fluorescence microscope (Olympus, Tokyo, Japan).

### Statistical analyses

All the data were analyzed via GraphPad Prism 10 statistical software. Two-tailed independent samples t-tests were implemented for multiple sets of data that met the normal distribution and chi-square, and Mann–Whitney U-tests were used to analyze between-group differences for data that did not meet the conditions. The results are shown as the means ± standard errors of the means (SEMs). Statistically significant differences were determined with a *P* value of less than 0.05, with significance levels indicated as follows: *P < 0.05, **P < 0.01, ***P < 0.001. “n” indicates the number of independent repetitions of the experiment.

## Results

### FKBP5 expression is significantly increased in the decidual tissue of OAPS patients

**Fig. 1:**
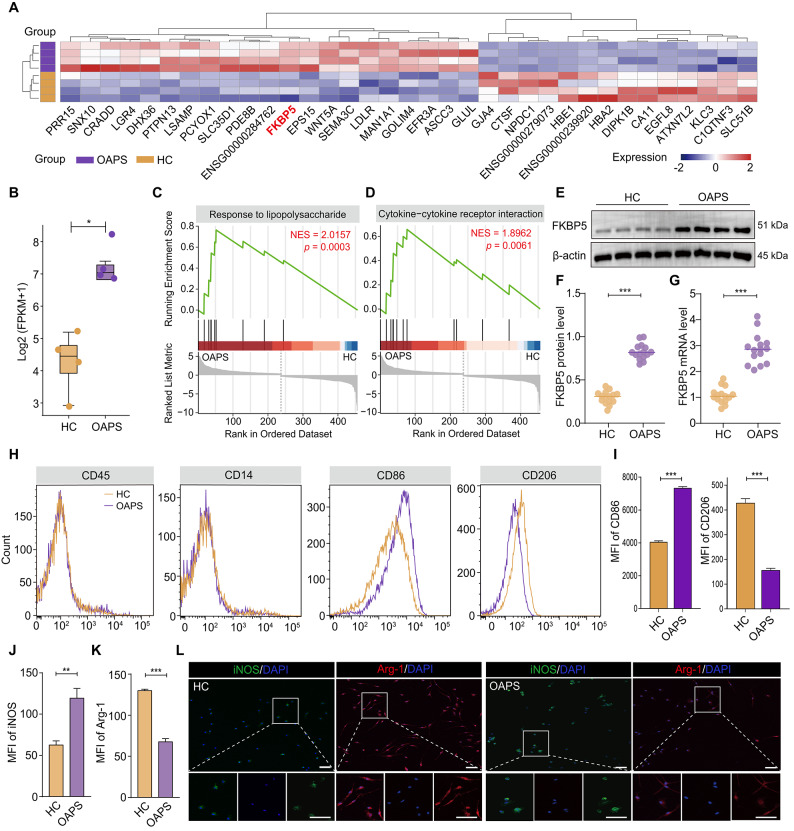
FKBP5 expression is significantly increased at the maternal‒fetal interface in patients with OAPS, accompanied by abnormal M1/M2-like macrophage polarization at this interface. (**A**) Heatmap of representative differentially expressed genes in decidual tissue from HCs (n = 4) and OAPS patients (n = 4). (**B**) FKBP5 gene expression was significantly upregulated in the OAPS group. (**C**‒**D)** GSEA revealed a stronger response profile to responses to LPS and cytokine‒cytokine receptor interaction pathways in the OAPS group. NES, normalized enrichment score. (**E**–**F**) Elevated FKBP5 protein expression in the decidual tissue of OAPS patients (n = 15) and its statistical analysis. (**G**) Upregulation of FKBP5 mRNA expression in decidual tissue from OAPS patients (n = 15). (**H**–**I**) Differences in the expression of CD45, CD14, CD86 and CD206 in samples of the HC and OAPS groups were detected by FCM, and its statistical analysis. (**J**‒**L**) Representative IF images and statistical results of M1/M2-like macrophages in the OAPS and HC groups after FCM sorting. Scale bar = 40 μm. The data are shown as the means ± SEMs. *P < 0.05, **P < 0.01, ***P < 0.001.

Decidual tissue samples were obtained from OAPS patients (n = 4) and HC individuals (n = 4) who chose to terminate their pregnancies for non-medical reasons. RNA-seq was used to assess the variations in gene expression between the decidual tissues of the OAPS and HC groups. Figure 1A presents a heatmap of the DEGs, with screening criteria for the high-expression group set at a fold change exceeding 1.5 or falling below 0.666, a *P* value less than 0.01, and a minimum fragments per kilobase of transcript per million mapped reads (FPKM) greater than 1. Among the DEGs, the FKBP5 gene was significantly highly expressed in the OAPS group than in the HC group (Figure 1B); this gene encodes the immunomodulatory protein FKBP51. GSEA revealed that the LPS response and cytokine‒cytokine receptor interaction were significantly enriched pathways (Figure 1C, D). Furthermore, GO functional classification—encompassing molecular function (MF), cellular component (CC), and biological process (BP) analysis—combined with KEGG pathway enrichment analysis, revealed potential differences in disease-related biological functions between the two groups (Figure S3).

On the basis of the integrated analysis of gene expression levels from transcriptome data and continuous functional evaluations, we selected the FKBP5 gene as the focus of subsequent studies. We collected an additional 15 samples from both the OAPS group and the HC group and isolated decidual tissues to assess FKBP5 expression at the protein and mRNA levels through Western blotting and RT‒qPCR. The findings illustrated in Figure 1E–G were largely consistent with those of the RNA-seq analysis; specifically, the protein and transcript levels of FKBP5 in the OAPS group were nearly three times greater than those in the HC group. This finding preliminarily indicates the differential expression pattern of FKBP5 between the OAPS and HC groups. These findings suggest that FKBP5 may play a role in the immunopathogenesis of OAPS and deserve further investigation.

### Abnormal polarization of M1/M2-like macrophages may be present at the maternal‒fetal interface in patients with OAPS

FKBP5 plays a crucial immunomodulatory role in human pregnancy. Given that human decidual macrophages (dMφ) represent the predominant innate immune subset, we isolated and compared dMφ subpopulations from OAPS patients and the HC group via FCM. The FCM results revealed no statistically significant differences in the proportion or number of CD45^+^/CD14^+^ cells between the OAPS and HC groups. The abnormal polarization of M1/M2-like macrophages is implicated in the pathogenesis of various autoimmune diseases and pathological pregnancies of placental origin. Further, M1 (CD14^+^CD86^+^) and M2 (CD14^+^CD206^+^) macrophages were sorted based on M1/M2 polarization phenotypic markers. Notably, our analysis revealed that the proportion and quantity of M1 (CD14^+^CD86^+^) macrophages were significantly greater in the OAPS group, whereas those of M2 (CD14^+^CD206^+^) macrophages were notably lower (Figure 1H, I). These findings indicate that M1/M2-like macrophage polarization may play a role in the pathophysiology of OAPS. To further corroborate these findings, we assessed the differences in the expression of inducible nitric oxide synthase (iNOS), an M1 macrophage marker, and arginase-1 (Arg-1), indicative of M2 macrophages^+^ between the two groups via IF staining of FCM-sorted macrophages from patients and controls. The findings indicated that the proportion of M1 macrophages in the OAPS group was approximately doubled compared with the HC group, whereas the proportion of M2 macrophages was approximately halved (Figure 1J–L). These results preliminarily suggest that the maternal‒fetal interface in patients with OAPS may exhibit abnormal M1/M2 macrophage polarization.

### FKBP5 may promote M1 macrophage polarization and inhibit M2 macrophage polarization

**Fig. 2:**
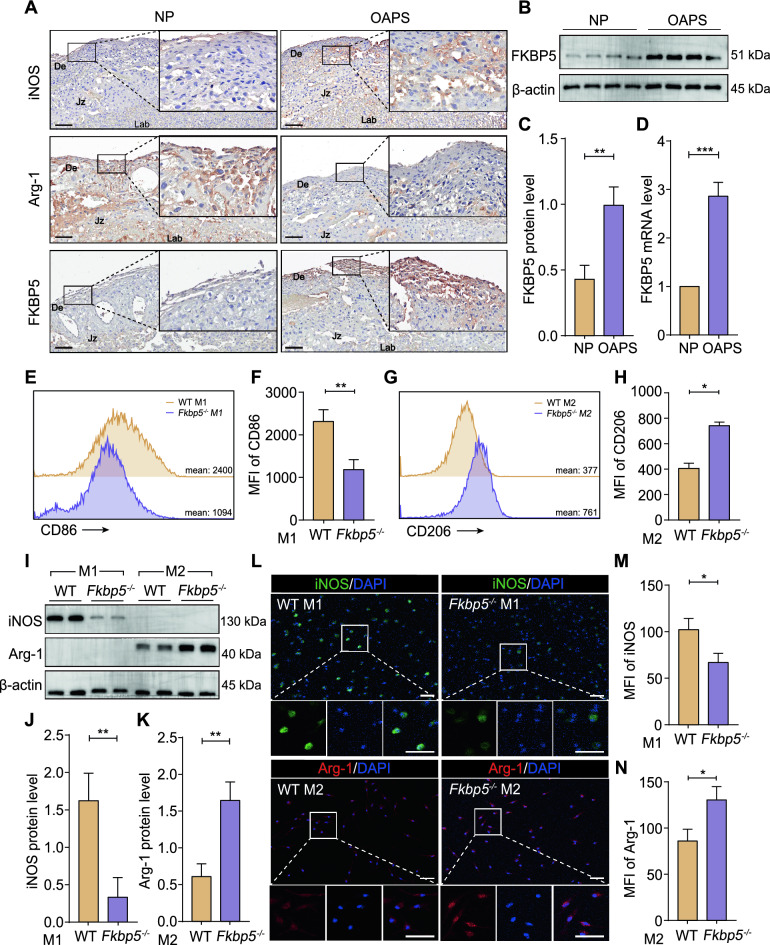
FKBP5 may promote M1 macrophage polarization and inhibit M2 macrophage polarization. (**A**) IHC staining showing the expression levels of iNOS, Arg-1 and FKBP5 at the placental interface in NP and OAPS mice. Scale bar = 100 μm. (**B**–**C**) Expression levels and statistical analysis of FKBP5 protein in the samples of mice from the NP and OAPS groups. (**D)** Increased expression of FKBP5 mRNA in samples from the OAPS mouse model. (**E**–**H**) Representative FCM data of M1 macrophages (CD86^+^) in both the WT M1 group and the *Fkbp5*^*-/-*^ M1 group, M2 macrophages (CD206^+^) in the WT M2 group and the *Fkbp5*^*-/-*^ M2 group and their statistics in the in vitro macrophage induction models of BMDMs. (**I**‒**K**) Western blotting showing changes and statistical analyses of protein expression levels of M1 macrophages (iNOS^+^) in the WT M1 group and the *Fkbp5*^*-/-*^ M1 group, as well as of M2 macrophages (Arg-1^+^) in the WT M2 group and the *Fkbp5*^*-/-*^ M2 group, within the in vitro induction model of BMDMs. (**L**‒**N**) IF detection of representative images of M1 macrophages (iNOS^+^) in both the WT M1 and *Fkbp5*^*-/-*^ M1 groups and M2 macrophages (Arg-1^+^) in the WT M2 and *Fkbp5*^*-/-*^ M2 groups from the in vitro induction model of BMDMs; the results were statistically analyzed. Scale bar = 40 μm. The data are shown as the means ± SEMs. *P < 0.05, **P < 0.01, ***P < 0.001.

To investigate the association between heightened FKBP5 expression in the OAPS mice model and dysregulated M1/M2 macrophage polarization, we established comparative cohorts of OAPS-model mice and NP- control mice (refer to the Methods section). First, we performed IHC staining to assess the expression levels of iNOSArg-1 in the placental tissues of both the OAPS and the NP groups, alongside the expression of the FKBP5 protein in these tissues. Figure 2A shows that iNOS levels were significantly greater in the placentas of the OAPS group than in those of the NP group, whereas Arg-1 expression was lower. Notably, the FKBP5 expression pattern in the placentas of OAPS mice was consistent with that of iNOS, in contrast with the expression pattern of Arg-1. Macrophages were isolated from whole placental tissue containing the decidual interface and assessed FKBP5 protein and transcript levels through Western blotting and RT‒qPCR. The results indicated that FKBP5 expression in the placental tissue of OAPS mice was significantly elevated by 2‒3 fold compared with that in the NP group (Figure 2B–D). These findings suggest that FKBP5 is highly expressed at the maternal‒fetal interface and may contribute to the pathological processes of OAPS by influencing the abnormal polarization of macrophages.

To elucidate the relationship between FKBP5 and abnormal polarization of macrophages, we constructed an in vitro macrophage induction model utilizing FKBP5 knockout mice and WT mice. BMDMs (M0) were obtained from both groups of mice, followed by induction of M1 polarization with LPS and IFN-γ, and M2 polarization with IL-4 and IL-13. This approach created four distinct subgroups, the WT M1 group, the *Fkbp5*^*-/-*^ M1 group, the WT M2 group, and the *Fkbp5*^*-/-*^ M2 group, which were utilized to evaluate how FKBP5 knockdown affects the polarization of primary macrophages. We first evaluated the expression of macrophage subtypes via FCM, with CD86 and CD206 serving as markers for M1 and M2 macrophages, respectively. The expression of CD86^+^ M1-like macrophages in the *Fkbp5*^*-/-*^ M1 group was approximately 0.5 times lower than that in the WT group, whereas the expression of CD206^+^ M2-like macrophages in the *Fkbp5*^*-/-*^ M2 group was approximately twofold higher than that in the WT group (Figure 2E–H). We subsequently examined the expression levels of marker proteins following M1/M2 polarization of macrophage via Western blotting. The findings revealed that iNOS levels were approximately 0.25 times lower in the *Fkbp5*^*-/-*^ M1 group than in the WT M1 group, whereas Arg-1 levels were approximately three times higher in the *Fkbp5*^*-/-*^ M2 group than in the WT M2 group (Figure 2I–K). Additionally, we assessed the polarization status of four groups of macrophages after induction by IF. As shown in Figure 2L–N, after knockdown of FKBP5, the mean fluorescence intensity of iNOS^+^ in the *Fkbp5*^*-/-*^ M1 group was significantly lower than that in the WT M1 group, whereas the mean fluorescence intensity of Arg-1^+^ in the *Fkbp5*^*-/-*^ M2 group was significantly greater than that in the WT M2 group.

The results from both the animal model and the in vitro induced cell model indicate that FKBP5 may play a important role in regulating macrophage polarization. Specifically, FKBP5 may promote the polarization of M1 macrophages while inhibiting the polarization of M2 macrophages.

### FKBP5 may regulate macrophage polarization through the JAK-STAT pathway

**Fig. 3:**
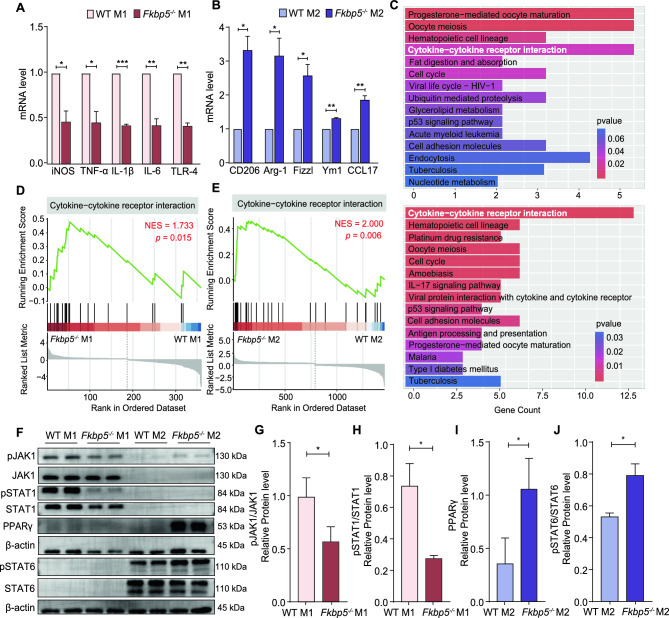
FKBP5 may regulate macrophage polarization via the JAK‒STAT pathway. (**A**–**B**) The mRNA levels of M1 macrophage markers (iNOS, TNF-α, IL-1β, IL-6, and TLR-4) in both the WT M1 and *Fkbp5*^*-/-*^ M1 groups and M2 macrophage markers (CD206, Arg-1, Fizzl, Yml, and CCL17) in the WT M2 and *Fkbp5*^*-/-*^ M2 groups were determined via RT‒qPCR. (**C**) Functional enrichment analysis of the WT M1 and *Fkbp5*^*-/-*^ M1 groups and the WT M2 and *Fkbp5*^*-/-*^ M2 groups via KEGG pathway analysis. (**D**–**E**) GSEA revealed a stronger response to the cytokine‒cytokine receptor interaction pathway in terms of both M1 macrophage polarization (WT M1 group and *Fkbp5*^*-/-*^ M1 group) and M2 macrophage polarization (WT M2 group and *Fkbp5*^*-/-*^ M2 group). (**F**‒**J**) Western blotting results showing representative protein bands and statistical values of pJAK1/JAK1, pSTAT1/STAT1, PPARγ and pSTAT6/STAT6 in the four groups of BMDMs: WT M1, *Fkbp5*^*-/-*^ M1, WT M2 and *Fkbp5*^*-/-*^ M2. The data are shown as the means ± SEMs. *P < 0.05, **P < 0.01, ***P < 0.001.

To deeply explore the mechanism of FKBP5 regulation of macrophage polarization, we used in vitro cell induction models of WT and *Fkbp5*^*-/-*^ mouse BMDMs. We next assessed the differences in the transcript levels of M1/M2 macrophage marker genes following FKBP5 knockdown through RT‒qPCR. The results indicated that the expression levels of genes such as the iNOS, TNF-α, IL-1β, IL-6, and TLR-4 genes in the *Fkbp5*^*-/-*^ M1 group were approximately 0.5 times lower than that of the WT M1 group. (Figure 3A). Conversely, the expression levels of genes, including the CD206, Arg-1, Fizzl, Ym1, and CCL17 genes, in the *Fkbp5*^*-/-*^ M2 group were approximately 2–3 times greater than those in the WT M2 group (Figure 3B). These results not only suggest that FKBP5 can regulate M1 and M2 macrophage polarization at the transcriptional level, but also provide an experimental basis for the next step of using the transcriptome to explore the mechanism of FKBP5 regulating macrophage polarization.

Total RNA was collected from the four aforementioned groups of cells, and RNA-seq was utilized to identify DEGs and associated signaling pathways. Transcriptome heatmaps, volcano plots, and GO functional classification analyses revealed distinct differences across the groups, with the majority of the DEGs closely linked to inflammatory responses and immune regulation (Figure S4). Furthermore, KEGG and GSEA revealed the involvement of multiple signaling pathways in FKBP5-mediated regulation of M1/M2 macrophage polarization. Notably, the cytokine‒cytokine receptor interaction signaling pathway was found to be related to both M1 and M2 macrophage polarization processes, with significant differences (Figure 3C–E). The primary downstream pathway of the cytokine‒cytokine receptor interaction pathway is the JAK‒STAT pathway^33^; aberrant activation of this pathway has been shown to result in an imbalance between macrophage subtypes, potentially triggering or exacerbating inflammatory responses. We subsequently assessed the expression levels of target proteins in the JAK‒STAT pathway across the WT M1 group, *Fkbp5*^*-/-*^ M1 group, WT M2 group, and *Fkbp5*^*-/-*^ M2 group to clarify the molecular mechanisms by which FKBP5 influences macrophage polarization. Western blotting results indicated that the pJAK1/JAK1 in the WT M1 group was approximately twice greater than that in the *Fkbp5*^*-/-*^ M1 group, and the pSTAT1/STAT1 in the WT M1 group was approximately three times greater than that in the *Fkbp5*^*-/-*^ M1 group. PPARγ was approximately threefold higher in the *Fkbp5*^*-/-*^ M2 group than in the WT M2 group, and pSTAT6/STAT6 was approximately 1.5-fold higher in the *Fkbp5*^*-/-*^ M2 group than in the WT M2 group. (Figure 3F–J). These findings suggest that FKBP5 may promote M1 macrophage polarization via the JAK1/STAT1 pathway while inhibiting M2 macrophage polarization through the PPARγ/STAT6 pathway.

### FKBP5 inhibits trophoblast function through aberrant activation of macrophage polarization

**Fig. 4:**
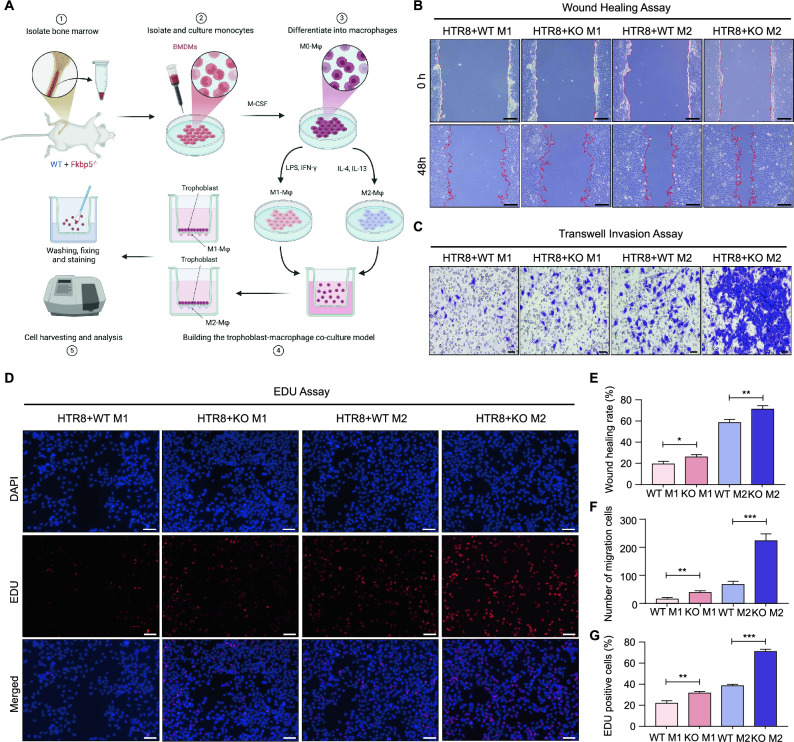
FKBP5 inhibits trophoblast function through aberrant activation of macrophage polarization. (**A**) Experimental flow chart for establishing the coculture system. BMDM (M0) were obtained from WT and *Fkbp5*^*-/-*^ mice, and M1 polarization was induced with LPS (100 ng/mL) and IFN-γ (20 ng/mL), and M2 polarization was induced with IL-4 (20 ng/mL) and IL-13 (10 ng/mL), and subsequently the supernatants of the above four groups of macrophages were used as conditioned medium to establish a co-culture system with HTR8/SVneo cells. (**B**) A wound healing assay was performed to assess the migration capacity of HTR8/SVneo cells in the four groups. Scale bar = 50 μm. (**C**) Transwell assays were used to evaluate the invasive ability of HTR8/SVneo cells in the four groups. Scale bar = 40 μm. (**D**) EDU was used to detect the proliferative capacity of HTR8/SVneo cells in the four groups. Scale bar = 40 μm. (**E**–**G**) Statistical analysis of the wound healing, transwell and EDU experiments, respectively. The data are shown as the means ± SEMs. *P < 0.05, **P < 0.01, ***P < 0.001.

Coordinated crosstalk between macrophages and trophoblasts is essential for maternal–fetal immune tolerance, as well as for placental development and functional maintenance^9,34^. To investigate the impact of abnormal polarization of M1/M2 macrophages on trophoblast function, we collected supernatants from cultured cells from four groups: WT M1, *Fkbp5*^*-/-*^ M1, WT M2, and *Fkbp5*^*-/-*^ M2. We subsequently established a coculture system with HTR8/SVneo cells to assess changes in the migratory, invasive, and proliferative capabilities of trophoblasts. The cocultured cells were designated the WT M1, KO M1, WT M2, and KO M2 groups, with the coculture pattern illustrated in Figure 4A.

We utilized the wound healing assay (indicative of migratory ability) to assess the migratory ability of trophoblasts (HTR8/SVneo) (Figure 4B, E), the Transwell assay to evaluate its invasive capacity (Figure 4C, F), and the EDU assay to measure its proliferative potential (Fig 4D, G). The results across the four groups exhibited similar trends. Compared with those in the WT M1 group, the wound healing rates, invasive capacity, and proliferative ability of HTR8/SVneo cells in the WT M2 group were approximately 39%, threefold, and 16% greater, respectively, suggesting that M2 macrophage polarization enhances trophoblast function. Additionally, when the migratory, invasive, and proliferative capacities of the KO M1 were increased by approximately 7%, twofold, and 10%, respectively, compared with those of the WT M1. These findings indicate that FKBP5 knockdown results in reduced M1 macrophage polarization, and that the function of trophoblast cells is significantly improved after constructing the co-culture model. Furthermore, compared with the WT M2 group, the KO M2 group presented more significant enhancements in migration, invasion, and proliferation capacities of approximately 13%, threefold, and 30%, respectively. These findings suggest that FKBP5 knockdown promotes macrophage M2-like polarization and significantly improves functional damage of trophoblast cells after establishing a co-culture model.

Above results suggest that FKBP5 may drive macrophage polarization toward M1-like and inhibit M2-like polarization, leading to an imbalance in the immune microenvironment at the maternal–fetal interface, which in turn reduces the migration, invasion, and proliferative activity of trophoblast cells. This coordinated interaction between functional cells at the maternal–fetal interface may be an important molecular mechanism of pathological pregnancy in OAPS.

### FKBP5 causes adverse pregnancy outcomes in OAPS mice model

**Fig. 5:**
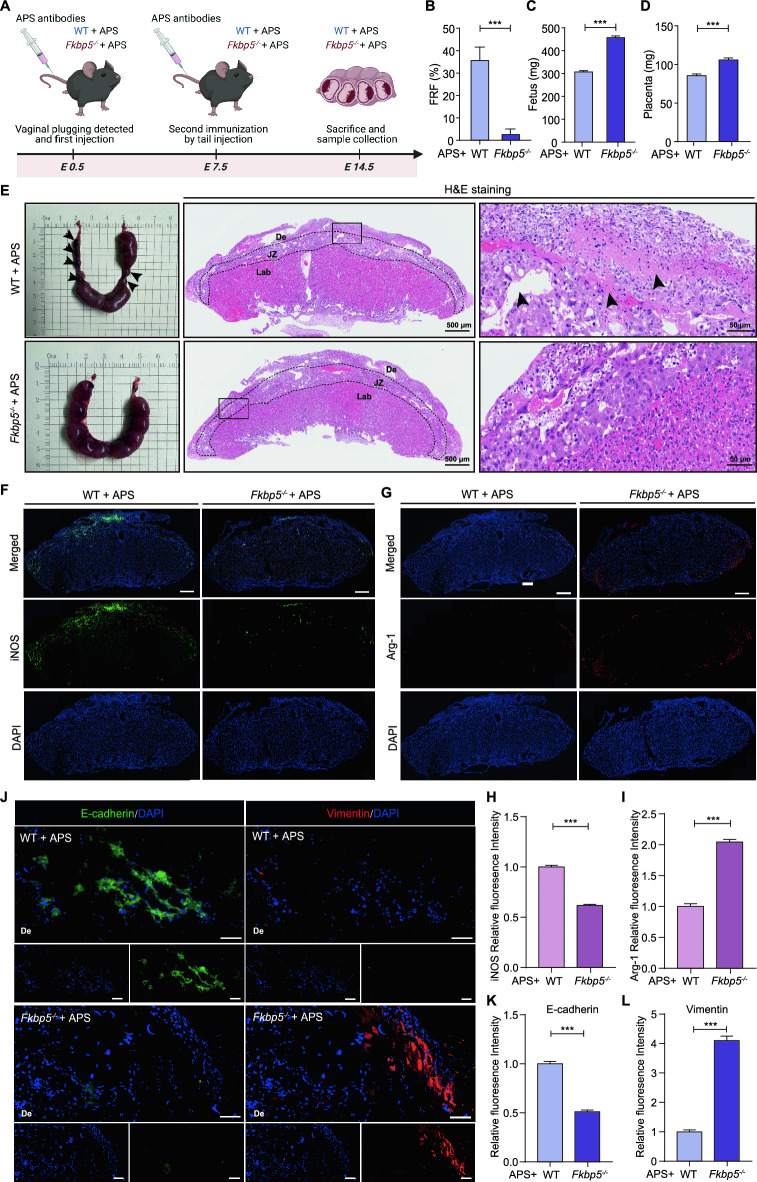
FKBP5 causes adverse pregnancy outcomes in OAPS mice model. (**A**) Schematic representation of the establishment of the WT + APS and *Fkbp5*^*-/-*^ + APS mouse models. (**B**–**D**) FRF, mean fetal weight and mean placental weight were compared between the WT and *Fkbp5*^*-/-*^ groups of mice at E14.5 and analyzed statistically. The FRF is calculated as the number of fetuses absorbed divided by the total number of fetuses (the number of fetuses absorbed plus the number of surviving fetuses). (**E**) Representative images of uterine morphology and placental HE from the WT + APS and *Fkbp5*^*-/-*^ + APS groups. De, decidua basalis; Jz, junctional zone; Lab, labyrinth. The black arrows in the mouse uterine morphology image indicate stillbirths, placental hematomas, and fetal growth restrictions observed in the mice; the black arrows in the HE staining image indicate areas of placental tissue necrosis and vascular lesions. (**F**–**I**) Changes in the fluorescence intensities of iNOS and Arg-1 at the placental interface in the WT + APS and *Fkbp5*^*-/-*^ + APS groups before and after FKBP5 knockdown were compared and statistically analyzed. Scale bar = 500 μm. (**J**–**L**) Representative fluorescence images of E-cadherin and Vimentin at the placental interface of mice in both the WT + APS and *Fkbp5*^*-/-*^ + APS groups and statistical results. Scale bar = 50 μm. The data are shown as the means ± SEMs. *P < 0.05, **P < 0.01, ***P < 0.001.

To further clarify the mechanism of FKBP5 in regulating macrophage polarization at the maternal–fetal interface involved in aPLs-induced pathological pregnancy of placental origin, we used anti-β_2_GP1 antibody to construct OAPS in FKBP5 knockout mice (*Fkbp5*^*-/-*^ + APS group) and control mice (WT + APS group), respectively. This approach aimed to assess the damaging role of FKBP5 in OAPS pathologic pregnancies by comparing the differences in adverse pregnancy outcomes between two groups of OAPS-induced mouse models. The flow chart depicting the mouse modeling process is presented in Figure 5A.

The pregnancy outcome was assessed by calculating the FRF, mean fetal weight, and mean placental weight for each group. The statistical results indicated that the FRF in the WT + APS group was 35.6%, with a mean fetal weight of approximately 306.9 mg and a mean placental weight of approximately 85.5 mg. The FRF in the *Fkbp5*^*-/-*^ + APS group was 2.5%, with a mean fetal weight of approximately 456.8 mg and a mean placental weight of approximately 105.9 mg (Figure 5B–D, Figure S5A). Compared with those in the WT + APS group, the FRF in the *Fkbp5*^*-/-*^ + APS group was 33.1% lower, the average fetal weight was 48.8% greater, and the average placental weight was 23.9% greater. Furthermore, the placental blood supply in the *Fkbp5*^*-/-*^ + APS group was increased, which may have contributed to a notable decrease in the incidence of conditions such as early embryo loss and fetal growth restriction (Figure S5B). Next, we tested the differences in placental structure between the two groups via hematoxylin and eosin (HE) staining. Compared with those in the WT + APS group, the lesions at the placental interface in the *Fkbp5*^*-/-*^ + APS group transformed from stripes or sheets to dots, with fewer affected blood vessels. Pathological damage, such as vessel wall degeneration, degradation, or collapse of the vascular lumen, was also attenuated (Figure 5E). These findings indicate that the knockdown of FKBP5 can improve pregnancy outcomes and alleviate placental injury in OAPS.

To explore the mechanism by which FKBP5 improves pregnancy outcomes, we utilized IF staining to assess the expression levels of iNOS and Arg-1 in the placental tissues of the two groups of mice. Compared with the WT + APS group, there was a significant decrease in M1 macrophages (iNOS^+^) and a significant increase in M2 macrophages (Arg-1^+^) at the placental interface in the *Fkbp5*^*-/-*^ + APS group (Figure 5F–I). It suggests that FKBP5 deficiency may significantly promotes macrophage M2-like polarization and inhibits M1-like polarization. In addition, EMT is a biological phenomenon characterized by the loss of epithelial characteristics and the acquisition of mesenchymal properties, which are essential for a successful pregnancy. Abnormalities in this process can lead to poor pregnancy outcomes^35^. The regulatory factors E-cadherin and Vimentin are indicators of the EMT process. As depicted in Figure 5J–L, E-cadherin levels in the *Fkbp5*^*-/-*^ + APS group were significantly lower than those in the WT + APS group, whereas Vimentin levels were considerably higher in the *Fkbp5*^*-/-*^ + APS group than in the WT + APS group. This suggests that FKBP5 absence may promote the EMT process and improves maternal–fetal interface cell function.

Using the above aPLs-induced animal model, it was preliminarily found that FKBP5 deficiency could promote M2-like polarization of placental interface macrophages and inhibit M1-like polarization to remodel the immune microenvironment at the maternal–fetal interface; as well as enhance the plasticity of placental functional cells by improving EMT, which could in turn reverse aPLs-induced pregnancy injury. This finding provides an important experimental basis for the development of therapeutic strategies targeting FKBP5.

### In vivo inhibition of FKBP5 mitigates aPLs-induced maternal‒fetal interface damage

**Fig. 6:**
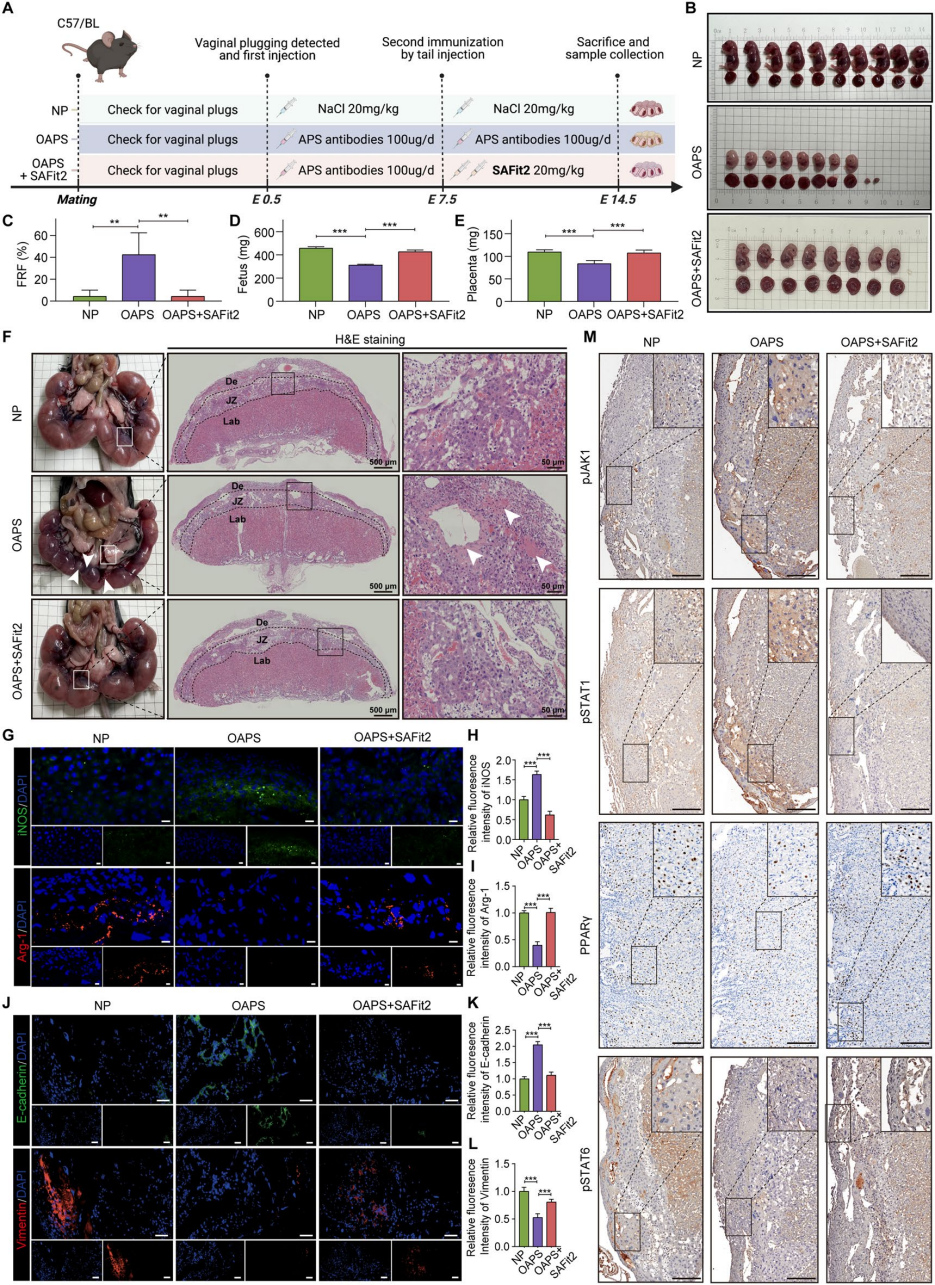
In vivo inhibition of FKBP5 mitigates aPLs-induced maternal‒fetal interface damage. (**A**) Schematic diagram of the experimental protocol for constructing the NP, OAPS, and OAPS + SAFit2 mouse models. (**B**–**E**) Representative images of E14.5 uterine morphology in the NP, OAPS and OAPS + SAFit2 groups of mice. FRF, mean fetal weight and mean placental weight were compared among the three groups of model mice. (**F**) Representative photographs of uterine morphology and histologic analysis of placentas from the NP, OAPS, and OAPS + SAFit2 groups. The white arrows indicate instances of stillbirths, placental hematomas, and fetal growth restriction in mice or areas of necrotic placental tissue and diseased blood vessels. (**G**–**I**) IF showing changes in the expression of iNOS and Arg-1 at the placental interface among the NP, OAPS, and OAPS + SAFit2 groups, which were statistically analyzed. Scale bar = 20 μm. (**J**‒**L**) Representative IF images of E-cadherin and Vimentin at the placental interface across the NP, OAPS, and OAPS + SAFit2 groups, which were statistically analyzed. Scale bar = 50 μm. (**M**) IHC revealed altered localization and expression of pJAK1, pSTAT1, PPARγ, and pSTAT6 at the placental interface in the NP, OAPS, and OAPS + SAFit2 groups of mice. Scale bar = 100 μm. The data are shown as the means ± SEMs. *P < 0.05, **P < 0.01, ***P < 0.001.

There is a bottleneck in the treatment of OAPS^5^, and the aforementioned experimental results indicate that FKBP5 may serve as a potential therapeutic target for this condition. SAFit2, a sulfonamide analog, exhibits potent and highly selective binding to FKBP5^36^. To elucidate the therapeutic effects of FKBP5 inhibitors in the OAPS mouse model, we established three groups: control (NP), OAPS-induced (OAPS), and treatment (OAPS + SAFit2) groups. Mice in both the OAPS and OAPS + SAFit2 treatment groups received an injection of a custom anti-β_2_GP1 antibody at E0.5 and E7.5, respectively, following the detection of vaginal plug. Additionally, the mice in the treatment group were daily administered the FKBP5 inhibitor SAFit2 (20 mg/kg) from gestational days E8.5 to E13.5. The flowchart depicting the mouse modeling process is illustrated in Figure 6A.

Pregnancy outcomes and placental structures were compared between groups to evaluate whether SAFit2, a small-molecule inhibitor of FKBP5, could mitigate the detrimental impacts of anti-β_2_GP1 antibodies. First, pregnancy outcomes were assessed by calculating the FRF, mean fetal weight, and mean placental weight for each group separately. In the OAPS group, the FRF was 42.33%, the mean fetal weight was approximately 310.4 mg, and the mean placental weight was approximately 83.4 mg. Whereas the treatment group presented values of 4.22% for FRF, 426.6 mg for mean fetal weight, and 107.4 mg for mean placental weight (Figure 6B–E). Compared with the OAPS group, the treatment group presented a 38.11% decrease in FRF, a 37.4% increase in mean fetal weight, and a 28.8% increase in mean placental weight. (Figure S5C, D). HE staining was used to observe the differences in placental structure among the groups. The placental interface of the mice in the SAFit2-treated group presented a reduced extent of lesions, fewer diseased blood vessels, and a significant reduction in pathological injuries, such as degeneration, degradation, or lumen collapse (Figure 6F). These results are consistent with the pregnancy outcomes and placental characteristics observed in the *Fkbp5*^*-/-*^ + APS group of mice, suggesting that SAFit2 may ameliorate pathological pregnancy injuries in OAPS by inhibiting the damaging effects of FKBP5 on the placenta, thereby improving overall pregnancy outcomes.

The expression levels of iNOS and Arg-1 in placental tissues from mice across all groups were evaluated via IF staining. The results presented in Figure 6G–I indicate that the expression of iNOS at the placental interface in the OAPS group was significantly elevated, whereas Arg-1 expression was notably reduced; whereas, in the treatment group, SAFit2 was able to inhibit the expression of iNOS and promote the expression of Arg-1, and the aberrant polarization of macrophages at the placental interface was significantly improved. Furthermore, we examined the differences in the EMT process at the placental interface across the three groups of mice. Compared with the NP group, the OAPS group presented significant increases in E-cadherin expression and considerable decreases in Vimentin expression. Conversely, the FKBP5 inhibitor SAFit2 inhibited E-cadherin expression and promoted Vimentin expression, resulting in a shift in trophoblasts from an epithelial phenotype to a highly motile mesenchymal phenotype (Figure 6J–L). Finally, we evaluated key proteins in the JAK‒STAT pathway at the placental interface among the three groups of mice via IHC. We focused on pathway proteins identified in previous in vitro experiments: pJAK1 and pSTAT1, which are associated with the M1 polarization of macrophages, and PPARγ and pSTAT6, which are linked to M2 macrophage polarization. The results indicated that the levels of pJAK1 and pSTAT1 in the placentas of the OAPS group were significantly greater than that of the NP group, while SAFit2 was able to reduce the expression of pJAK1 and pSTAT1; whereas the expression of PPARγ and pSTAT6 was significantly lower than that of the NP group, and SAFit2 was able to increase the expression of PPARγ and pSTAT6, which was consistent with the trend of in vitro cellular experiments (Figure 6M).

**Fig. 7:**
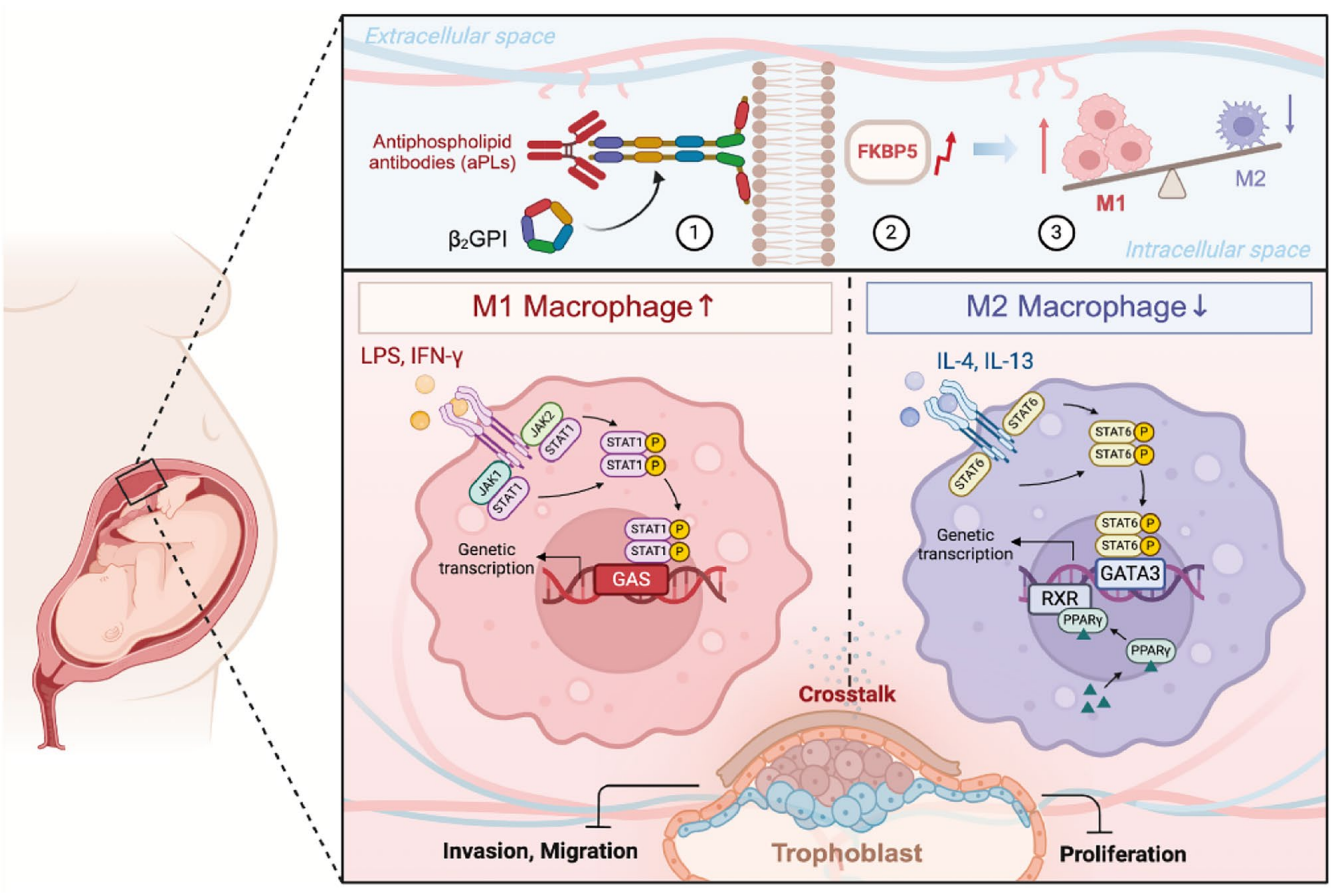
Schematic illustration of the involvement of FKBP5 in aPLs-induced mechanisms of functional impairment of trophoblast cells through the regulation of aberrant M1/M2 macrophage polarization at the maternal–fetal interface. Elevated FKBP5 expression at the maternal‒fetal interface in patients with OAPS may induce abnormal macrophage polarization, disturbing immune homeostasis and interfering with coordinated communication between functional cells. This disruption could impair the migratory, invasive, and proliferative abilities of trophoblasts, ultimately leading to adverse pregnancy outcomes.

These findings suggest that SAFit2 might serve as a promising therapeutic agent for OAPS. Figure 7 shows a schematic diagram depicting the mechanism by which FKBP5 may contribute to the pathogenesis of OAPS through the regulation of abnormal M1/M2 macrophage polarization. The aberrant polarization of decidual tissue macrophages disrupts immune homeostasis at the maternal‒fetal interface, while the impairment of trophoblast function, induced by aberrant intercellular crosstalk, may represent the fundamental molecular basis for pathological pregnancies of placental origin.

## Discussions

As a group of autoimmune diseases characterized by pregnancy-related complications, OAPS is a devastating “invisible killer” in the field of perinatal medicine, and its true burden of disease has been seriously underestimated. In this study, we revealed the immune role of FKBP5 in aPLs-induced placental injury through the regulation of macrophage polarization. These findings suggest a potential therapeutic target for OAPS.

The FKBP5 gene is a protein with a molecular weight of 51 kDa that is highly conserved across species. FKBP51 is involved in various physiopathological processes, including microtubule formation, cellular autophagy, steroid hormone receptor modulation, stress-related psychiatric disorders, and tumor regulation^37,38^. Studies have shown that FKBP5 is highly expressed in lymphocytes and has important immunoregulatory functions, participating in the activation and proliferation of T cells^39,40^. Our group recently discovered that FKBP5 reduces the expression of the Th17 cell-specific nuclear transcription factor RORγt through the inhibition of the AKT/mTOR signaling pathway, thereby inhibiting the differentiation of CD4^+^ T cells into Th17 cells. Additionally, we have conducted several studies on FKBP5 in the context of hormone-dependent reproductive disorders, suggesting its potential involvement in pathological pregnancy processes. Pregnancy is characterized by the precise regulation of various sex hormones, and the transcription of FKBP5 is influenced by these hormones. In addition to increasing the activity of the androgen receptor, FKBP5 also exerts an inhibitory effect on the activities of the progesterone receptor, estrogen receptor, and glucocorticoid receptor^41,42^. Our previous research indicated that reduced FKBP5 levels in endometrial adenocarcinoma tissues promote cancer cell proliferation by increasing p-AKT levels while simultaneously decreasing the sensitivity of these cancer cells to progesterone therapy. During decidualization, progesterone induces high expression of FKBP5, which significantly prolongs FOXO1A protein half-life by inhibiting phosphorylation at the AKT (Ser473) site, thereby driving decidualization of endometrial mesenchymal stromal cells and affecting the success of embryo transfer^20^. Due to the expression pattern of FKBP5 and its function in immunomodulation and hormone-dependent reproductive disorders, we hypothesized that this gene might be involved in the pathogenesis of OAPS, but to date, no reports have been published on the role of FKBP5 in the development of OAPS.

Decidual immune cells play a vital role in the establishment and maintenance of homeostasis in the maternal–fetal immune microenvironment. Studies targeting placenta-derived pathological pregnancies have found that aPLs-induced placental dysplasia may be predominantly immune-driven, including the release of neutrophil extracellular trapping networks (NETs)^43^, abnormalities in the phenotype and function of decidual macrophages^44^, functional activation of natural killer cells, and imbalance in the ratio of auxiliary T lymphocytes Th1/Th2^45^, which affects the function of trophoblast cells and interferes with pathophysiological processes such as embryo implantation and uterine spiral artery remodeling, which ultimately leads to early pregnancy failure and various pathological pregnancies in patients with OAPS^46,47^. Imbalance of immune homeostasis at the maternal–fetal interface permeates aPLs-induced placental injury^8^. Macrophages are highly plastic and play an important role in maternal–fetal immune tolerance and placental development in early pregnancy^48^, in which abnormal polarization of macrophages is one of the main mechanisms of pathological damage in placental failure. Notably, we observed an imbalance in the M1/M2 macrophage ratio at the maternal–fetal interface in patients with OAPS, with a significant increase in the proportion of pro-inflammatory M1-like macrophages and a significant decrease in the proportion of anti-inflammatory M2-like macrophages.

Much of the foundational research on aPLs-induced pathological pregnancies of placental origin has focused on the impairment of trophoblast and endothelial cell function^10,49^, with limited attention given to immune regulation. Our group previously demonstrated that aPLs can promote the release of NETs and impair the function of placental trophoblasts and endothelial cells^43^. A recent study utilizing scRNA-seq to elucidate the cellular landscape at the maternal‒fetal interface in OAPS revealed an imbalance between macrophage subsets in decidual tissues^45^. However, the mechanisms related to the immune aspects of OAPS remain underexplored. We conducted a preliminary investigation into the molecular mechanisms through which FKBP5 influences macrophage polarization. Analysis of RNA-seq data using an in vitro macrophage induction model in FKBP5 knockout mice revealed that the cytokine-cytokine receptor interaction pathway may be a key pathway in the regulation of macrophage polarization by FKBP5 involved in the pathogenesis of OAPS. When a cytokine binds to a receptor on the cell membrane, JAK kinase is activated and phosphorylates the receptor, which in turn phosphorylates the STAT protein. The phosphorylated STAT protein forms a dimer and enters the nucleus, affecting the transcription of specific genes and thus regulating specific biological functions of the cell^33^. Aberrant activation of JAK/STAT, the most prominent downstream pathway in the cytokine-cytokine receptor interaction pathway, is one of the major causes of macrophage polarization imbalance. Notably, in cases of renal injury, particularly those induced by unilateral ureteral obstruction (UUO) or ischemia‒reperfusion (IRI) injury, TREM2 deficiency exacerbates renal damage by promoting macrophage polarization toward the M1 proinflammatory phenotype via the JAK‒STAT pathway, ultimately reducing cell viability^50^. In a study examining magnetic cue-mediated bone repair, magnetic cues within the scaffold activated PPARγ receptors, leading to the downregulation of JAK‒STAT pathway members and increased fatty acid metabolism, which in turn promoted M2 macrophage polarization and increased cell proliferation and metabolism^51^. Our functional assays indicated that FKBP5 may facilitate the polarization of M1 macrophages through the JAK1/STAT1 pathway while inhibiting the polarization of M2 macrophages via the PPARγ/STAT6 pathway. These results may provide new perspectives on the immune mechanisms involved in the pathogenesis of OAPS.

The search for effective therapeutic agents for OAPS is a shared vision of all scientists in the perinatal field. In the present study, we preliminarily demonstrated that abnormal crosstalk between macrophages and trophoblasts leads to an imbalance in maternal–fetal immune tolerance, and that this immune microenvironmental disorder may be an important mechanism contributing to adverse pregnancy outcomes in patients with OAPS.

The specific role of FKBP5 within cells depends on the surrounding environment^52^. Stabilization of the estrogen receptor through the ERα/FKBP5 complex in breast cancer cells mediates endocrine therapy resistance^53^; whereas, in endometrial cancer, it exerts its anti-cancer function by degrading the proto-oncoprotein C-MYC protein through the ubiquitination pathway of the E3 ligase TRIM28^54^. This tissue specificity correlates with differences in the proteins with which it interacts. For example, in fat tissues FKBP5 synergizes with PHLPP to activate the PI3K/AKT pathway, driving PPARγ transcriptional activity and preadipocyte maturation^54^, whereas in colorectal cancers FKBP5 promotes NF-κB nuclear translocation through inhibition of IκBα degradation, which in turn upregulates MMP-2/MMP-9 expression and accelerates metastasis^17^. Notably, the pathologic regulatory network of FKBP5 has expanded to reproductive medicine. It has been shown that FKBP5 is significantly increased in placental tissues of patients with recurrent miscarriages, which is consistent with the high expression pattern of FKBP5 in decidual tissues of patients with OAPS in our study. Given the similarities between the immune microenvironment at the maternal–fetal interface and the tumor microenvironment in terms of immune metabolic reprogramming, hypoxic oxidative stress, etc., we hypothesized that FKBP5 may act as a “molecular switch” for immune homeostasis. FKBP5 can dynamically regulate the M1/M2 balance of macrophages and play an important role in the disruption of immune homeostasis at the maternal–fetal interface and pathological damage in OAPS patients.

The treatment of OAPS faces a significant limitation, as the currently employed antithrombotic therapy, which combines aspirin with low-molecular-weight heparin, is insufficient for treating refractory patients. Currently, HCQ is the only drug employed for experimental use in clinical settings. With increasing research into the mechanisms underlying OAPS, novel therapeutic agents, including low-dose glucocorticoids, immunosuppressive agents, pravastatin, and complement- and antibody-specific inhibitors, have been explored^25^. FK506 is used to treat autoimmune diseases by binding to FKBP5 through binding to the FKl structural domain^55^, which, along with azathioprine, is the only immunosuppressant allowed for use during pregnancy^56^. In a study that employed a β_2_GPI DNA vaccine to treat OAPS in mice, an adjuvanted vaccine combined with FK506 demonstrated significantly greater efficacy than the DNA vaccine alone, indicating that FK506 may have potential as a therapeutic agent for OAPS^57^. FK506, a potent ligand for FKBP5, may exert a therapeutic effect on OAPS mice by inhibiting the peptidyl-prolyl isomerase activity of FKBP5. These findings provide substantial evidence that FKBP5 may serve as a promising target for both the diagnosis and treatment of OAPS. Currently, there is a lack of functionally specific small-molecule therapeutic agents for the treatment of OAPS. SAFit2, a sulfonamide analog with FKBP5-binding properties, has emerged as a promising therapeutic target for stress-related psychiatric disorders and metabolic diseases and acts as a potent and highly selective FKBP5 inhibitor^58^. A study demonstrated that SAFit2, a highly selective inhibitor of FKBP5, improved the dysfunction of the hypothalamic‒pituitary‒adrenal (HPA) axis caused by chronic stress in mice^59^. Furthermore, SAFit2 promotes the phosphorylation of AS160, an AKT2 substrate involved in glucose uptake, increases GLUT4 expression and glucose uptake in order to effectively ameliorate type II diabetes phenotype^60^. We applied an animal model of OAPS and comprehensively evaluated the therapeutic effects of SAFit2 in OAPS. The results showed that SAFit2 could reverse the abnormal polarization of macrophage M1/M2 at the maternal–fetal interface, effectively improve the pathological damage of the placenta and adverse pregnancy outcome in OAPS, providing experimental support for the therapeutic potential of inhibiting FKBP5 in OAPS.

In conclusion, our study found that FKBP5 at the maternal–fetal interface regulates macrophage M1/M2 polarization, resulting in abnormal macrophage-trophoblast crosstalk in the immune microenvironment of the decidua, which may underlie the immune basis of aPLs-induced pathological injury of placenta origin. This provides a new idea to deeply elucidate the molecular mechanism of maternal–fetal immune tolerance in OAPS pathological pregnancies, and provides a new target for the treatment of OAPS.

The clinical translation of SAFit2 for OAPS confronts dual challenges: inherent limitations of FKBP5 mono-target inhibition, compounded by gestation-specific pharmacological vulnerabilities^61,62^. Beyond FKBP subtype heterogeneity and compensatory pathway risks, SAFit2’s established mechanisms—including HPA axis modulation (disrupting gestational glucocorticoid dynamics), insulin sensitization (compromising physiological insulin resistance), and sex hormone receptor crosstalk (via FKBP5-steroid interactions)—may perturb critical gestational adaptations essential for fetal development, glucose homeostasis, and placental immunotolerance. To overcome unresolved barriers—uncharacterized placental transfer kinetics, human FKBP5 subtype heterogeneity, and absent longitudinal immune data—a translational framework must prioritize: (1) Subtype-targeted nanodelivery to enhance specificity, (2) Rational combination therapies against resistance, (3) Human placental explant models for biodistribution validation, and (4) Large-cohort safety profiling addressing fetal programming effects. Only through such mechanistic precision can SAFit2 evolve from a promising inhibitor to a viable therapy for pregnant individuals, redefining targeted immunomodulation in high-risk gestation.

## Supplementary Information

Supplementary Information.

## Data Availability

The data that support the findings of this study are available from the corresponding author (Yu Xia, Department of Obstetrics and Gynecology, Shanghai Sixth People’s Hospital Affiliated to Shanghai Jiao Tong University School of Medicine, 600 Yishan Road, Shanghai 200233, China. obstrixia@gmail.com) upon reasonable request. The accession number for the RNA-seq data reported in this paper is GSE283798. Accession “GSE283798” is currently private and is scheduled to be released on Dec 31, 2025.
