# Supplementary material for: Inhibition of FKBP5 alleviates obstetric antiphospholipid syndrome by regulating macrophage polarization
Source: Sci Rep. 2025 Nov 21;15:41433. doi: 10.1038/s41598-025-25472-9 (PMC12638907; doi:10.1038/s41598-025-25472-9)
Supplement: Supplementary file 1 — Supplementary Information. [file 41598_2025_25472_MOESM1_ESM.pdf]

Primer sequences of genes for RT-qPCR analysis.

Genes	Forward 5'-3'	Reverse 5'-3'
Mice Genotype Identification		
Wild type mice	GAAGCCATTTGCCTTTAGCCTTG	GACTGTCATGATGCCCACGAG
Fkbp5 $^{-/-}$ *mice*	AATATGCCCCCTTGACTCTTTGCTG	GACTGTCATGATGCCCACGAG
Mouse		
Fkbp5	GACACCAAAGAAAAGCTGACG	CTTCTCTGACAGGCCGTATTC
β-actin	ACCTTCTACAATGAGCTGCG	CTGGATGGCTACGTACATGG
inos	GCAAACATCACATTCAGATCCC	TCAGCCTCATGGTAAACACG
Tnf-α	CTTCTGTCTACTGAACTTCGGG	CAGGCTTGTCACTCGAATTTTG
Il-1β	ACGGACCCCAAAAGATGAAG	TTCTCCACAGCCACAATGAG
Il-6	CAAAGCCAGAGTCCTTCAGAG	GTCCTTAGCCACTCCTTCTG
Tlr4	TTCAGAACTTCAGTGGCTGG	TGTTAGTCCAGAGAAACTTCCTG
Arg-1	AAGAATGGAAGAGTCAGTGTGG	GGGAGTGTTGATGTCAGTGTG
Cd206	ATGGATGTTGATGGCTACTGG	TTCTGACTCTGGACACTTGC
Fizz1	CGTGGAGAATAAGGTCAAGGAAC	CACACCCAGTAGCAGTCATC
Ym1	AGACTTGCGTGACTATGAAGC	ATGAATATCTGACGGTTCTGAGG
Ccl17	AGACCTTCACCTCAGCTTTTG	CTTTGAAGTAATCCAGGCAGC
Human		
Fkbp5	CAGTCTCCCTAAAATTCCCTCG	TTGCTCCTTCGTTTGGATTTG
β-actin	ACCTTCTACAATGAGCTGCG	CCTGGATAGCAACGTACATGG

Abbreviations: Tnf-α, Tumor Necrosis Factor-α; Tlr4, Toll-like Receptor 4; Fizzl, Found in Inflammatory Zone 1; Ccl17, C-C Motif Chemokine Ligand 17.

Figure S1. Primer sequences of genes for RT-qPCR analysis.

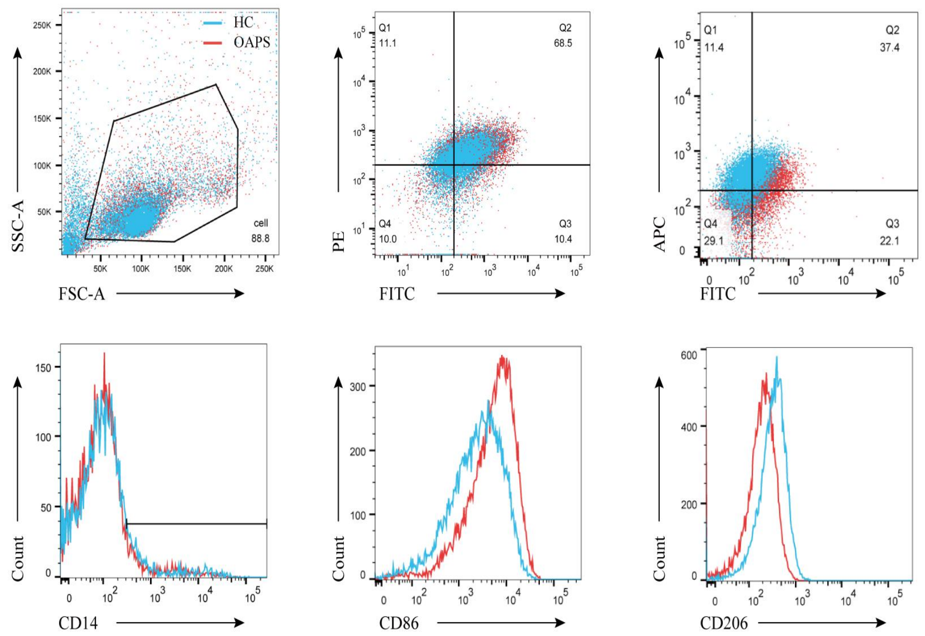

Figure S2. A representative image of flow cytometry gating strategy for decidual macrophages.

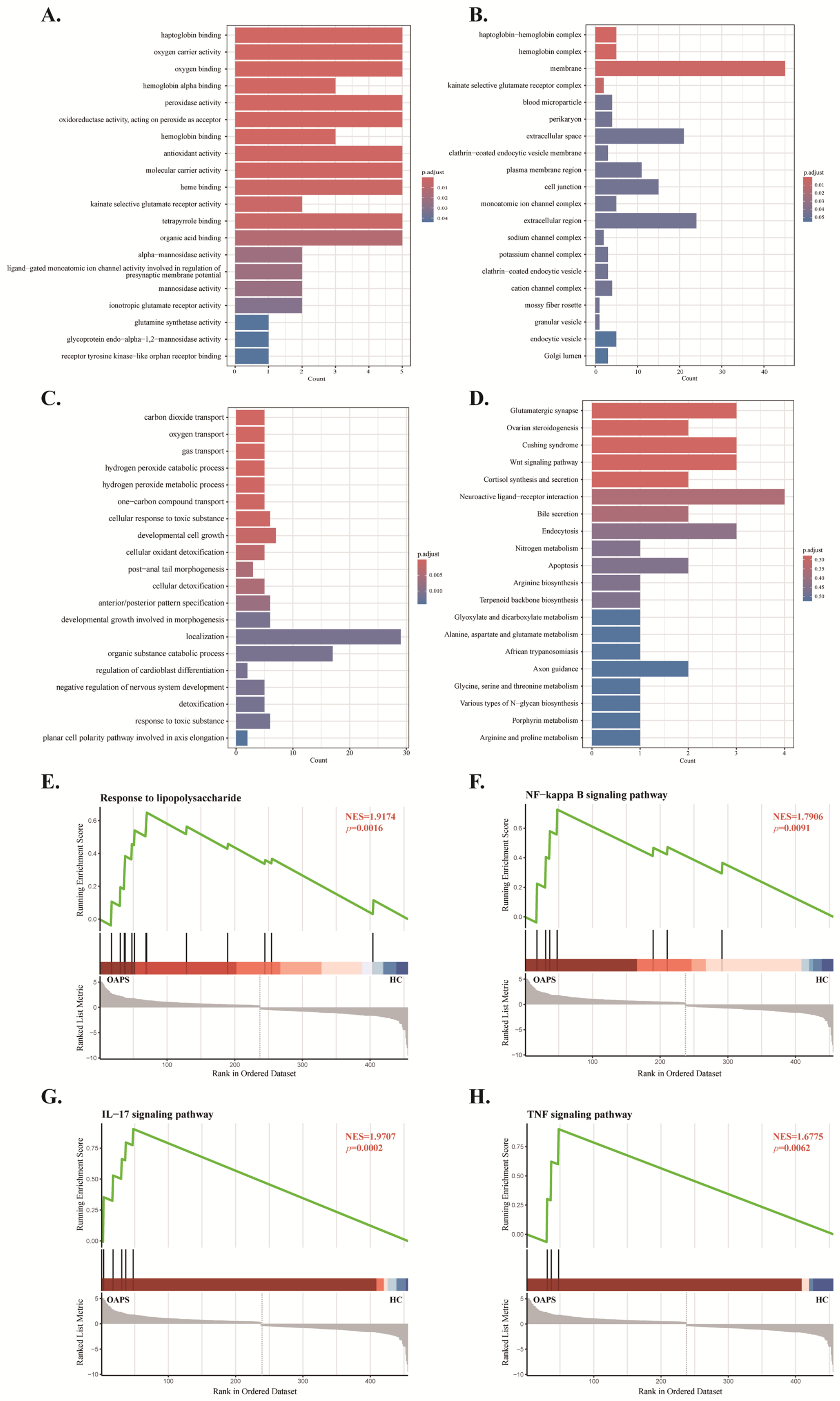

Figure S3. Supplementary images of RNA-seq in HC and OAPS patients.

A-D. GO functional classification covering MF, CC and BP was performed on HC and OAPS patients. **G-H.** The OAPS group responded more strongly to the GSEA analysis.

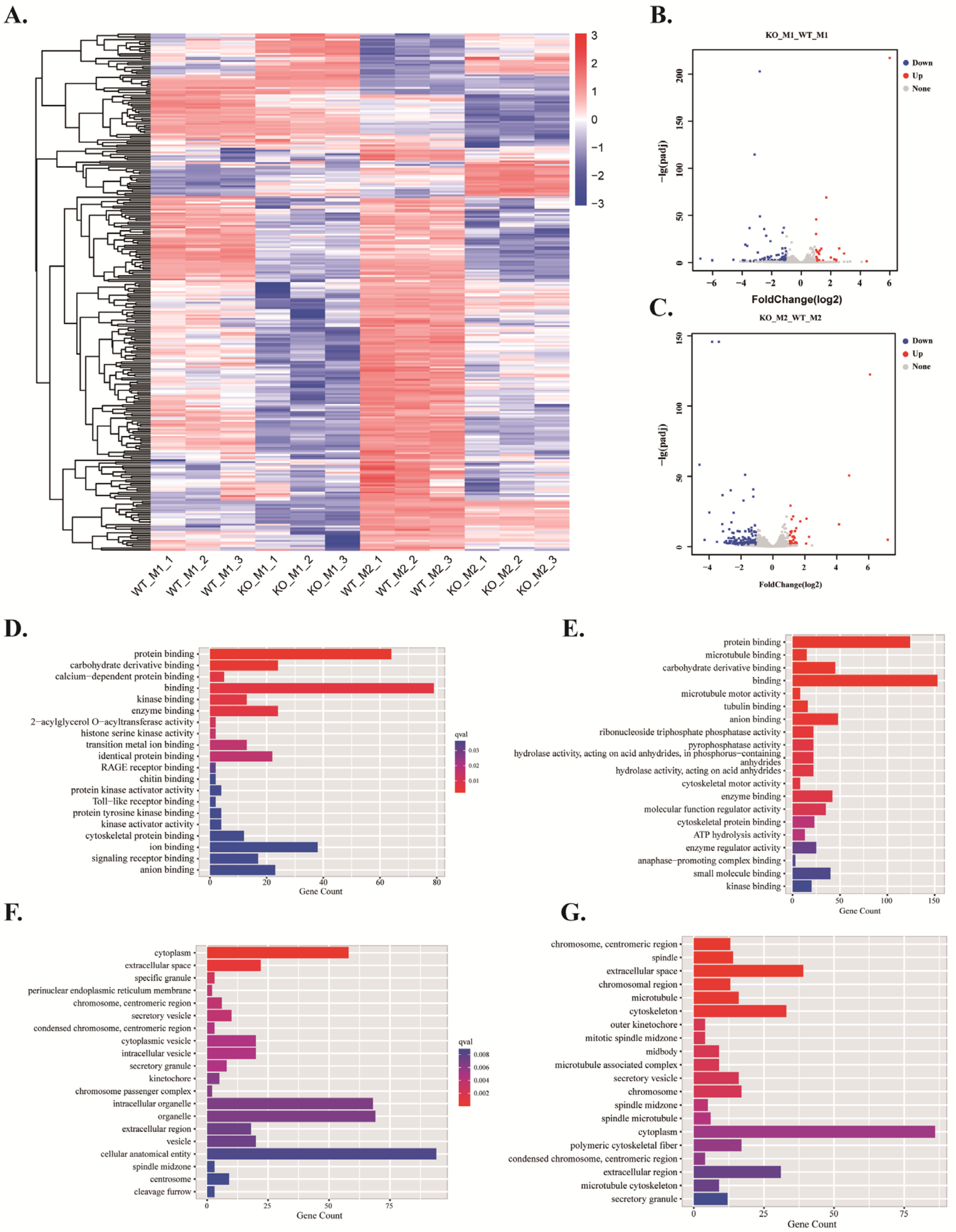

Figure S4. Supplementary images of in vitro-induced RNA-seq of four groups of BMDMs.

A. Heatmap of representative differential genes in the WT M1, *Fkbp5*$^{-/-}$ M1, WT M2 and *Fkbp5*$^{-/-}$ M2 groups. **B-C.** Volcano maps of four groups of BMDMs, WT M1, *Fkbp5*$^{-/-}$ M1, WT M2 and *Fkbp5*$^{-/-}$ M2. **D-G.** Four groups of BMDMs, WT M1, *Fkbp5*$^{-/-}$ M1, WT M2 and *Fkbp5*$^{-/-}$ M2, were analyzed for GO function covering MF, CC and BP.

A.

Therapeutic effects of FKBP5 knockout mice in an animal model of OAPS.

	FRF(%)	Fetus(mg)	Placenta(mg)
**WT+APS**	35.6±13.45	306.91±12.05	85.47±4.9
***Fkbp5*$^{-/-}$+APS**	2.5±5.59	456.82±18.34	105.86±5.71

B.

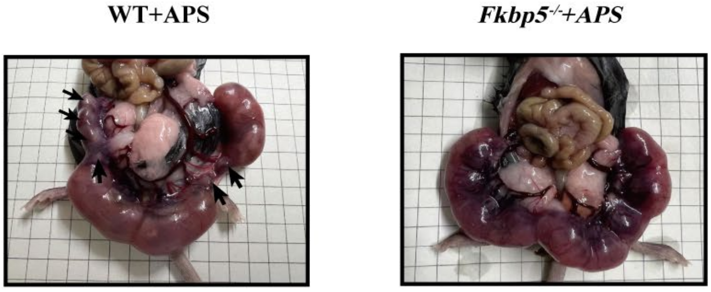

C.

Therapeutic effects of SAFit2 in an animal model of OAPS.

	FRF(%)	Fetus(mg)	Placenta(mg)
**NP**	2±5.79	456.8±13.27	109.6±4.88
**OAPS**	42.33±20.07	310.4±8.47	83.4±7.4
**OAPS+SAFit2**	4.22±5.79	426.6±15.08	107.4±6.5

D.

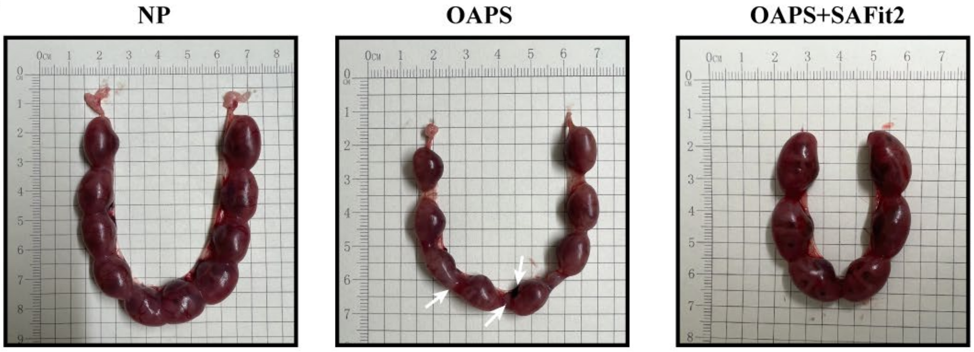

Figure S5. Supplementary data and images from in vivo experiments conducted in mice.

A. Therapeutic effects of FKBP5 knockout mice in an animal model of OAPS. **B.** Supplementary images of uterine morphology in the WT+APS and *Fkbp5*$^{-/-}$+APS groups of mice. Black arrows in mouse uterine morphology indicate stillbirths, placental hematomas, and fetal growth restriction observed in mice. **C.** Therapeutic effects of SAFit2 in the animal model of OAPS. **D.** Supplementary images of uterine morphology in the NP, OAPS, and treatment groups of mice. White arrows indicate observed stillbirths, placental hematomas, and fetal growth restriction in mice.
